# Supplementary material for: Clinical and Biological Adaptations in Obese Older Adults Following 12-Weeks of High-Intensity Interval Training or Moderate-Intensity Continuous Training
Source: Healthcare (Basel). 2022 Jul 20;10(7):1346. doi: 10.3390/healthcare10071346 (PMC9315493; doi:10.3390/healthcare10071346)
Supplement: Supplementary file 1 [file healthcare-10-01346-s001.zip › healthcare-1813078-supplementary.pdf]

Table 1: Delta changes of functional capacities and skeletal muscle functions in obese older adults following 12 weeks of High-Intensity Interval Training (HIIT) and Moderate-Intensity Continuous Training (MICT).

Parameters	HIIT (n=34)	MICT (n=34)	p-value
Functional capacities			
6 min walking test (m)	12.35 ± 12.97	5.21 ± 5.35	**0.005**
Step test (n)	17.02 ± 9.91	5.91 ± 15.00	**0.0007**
4 m walk test normal (m/s)	7.89 ± 10.69	6.44 ± 10.12	0.57
4 m walk test fast (m/s)	9.27 ± 9.89	8.47 ± 23.83	**0.85**
Balance test (s)	71.95 ± 112.49	51.10 ± 135.84	0.49
Chair test (s)	-17.04 ± 11.70	-4.65 ± 19.91	**0.002**
Timed Up and Go Test (s)	-7.47 ± 15.67	-7.69 ± 13.09	0.95
Skeletal muscle function			
Hand grip strength (kg)	12.28 ± 43.30	4.77 ± 19.87	0.38
Hand grip strength/BW	11.78 ± 42.32	5.61 ± 20.88	0.46
Hand grip strength/ALM	14.33 ± 42.82	8.24 ± 25.27	0.50
Quadriceps strength (N)	4.25 ± 18.44	23.17 ± 21.17	**0.0005**
Quad/BW	4.07 ± 18.44	23.62 ± 22.36	**0.0005**
Quad/LLM	2.28 ± 18.66	25.25 ± 25.23	**0.0001**
Lower limb power (W)	25.24 ± 28.05	20.42 ± 27.63	0.50

Data are presented as: mean ± SD. HIIT = high-intensity interval raining; MICT = moderate-intensity continuous raining; BW = body weight; ALM = arms lean mass; LLM = legs lean mass

Table S2: Delta changes of body composition parameters in obese older adults following 12 weeks of High-Intensity Interval Training (HIIT) and Moderate-Intensity Continuous Training (MICT)

Parameters	HIIT (n=34)	MICT (n=34)	p-value
	Anthropometry		
Weight (kg)	0.24 ± 2.48	-0.58 ± 4.32	0.33
BMI (kg/m^2)	0.17 ± 2.46	-0.58 ± 4.32	0.37
	Fat and lean mass (DXA)		
Total lean mass (kg)	1.58 ± 3.25	-0.81 ± 2.69	**0.002**
Arm lean mass (kg)	-0.15 ± 6.99	-2.30 ± 14.57	0.44
Leg lean mass (kg)	2.08 ± 4.22	-0.84 ± 5.16	**0.01**
Total fat mass (%)	-1.05 ± 5.08	-1.22 ± 4.50	0.89
Arm fat mass (%)	-2.77 ± 7.50	5.05 ± 8.48	**0.0001**
Leg fat mass (%)	-1.63 ± 6.63	-3.65 ± 5.66	0.19
Android fat mass (%)	-0.67 ± 5.44	-1.00 ± 7.72	0.84
Gynoid fat mass (%)	-1.09 ± 7.62	-4.20 ± 7.10	0.09
	Muscle composition (pQCT)		
Total muscle area (cm^2)	0.58 ± 10.41	-13.91 ± 17.94	**0.0004**
Total fat area (cm^2)	-0.12 ± 16.75	-9.72 ± 16.44	**0.03**
Subcutaneous fat area (cm^2)	1.15 ± 19.65	-10.93 ± 17.11	**0.01**
Intramuscular fat area (cm^2)	0.57 ± 62.12	16.41 ± 85.28	0.34

Data are presented as: mean ± SD. HIIT = high-intensity interval training; MICT = moderate-intensity continuous training; DXA = dual-energy X-ray absorptiometry; pQCT = peripheral quantitative computed tomography.

Table S3: Delta changes of blood parameters in obese older adults following 12 weeks o High-Intensity Interval Training (HIIT) and Moderate-Intensity Continuous Training (MICT)

Parameters	HIIT (n=34)	MICT (n=34)	p-value
	Blood Parameters		
Adiponectin (μg.ml^{-1})	-0.64 ± 32.64	-1.96 ± 13.04	0.83
Leptin (ng.ml^{-1})	33.35 ± 114.61	12.71 ± 70.85	0.39
Adiponectin/leptin	0.46 ± 57.88	6.50 ± 39.85	0.63
Free fatty acids (mmol.l^{-1})	2.60 ± 50.84	22.09 ± 70.20	0.20
Total cholesterol (mmol.l^{-1})	-0.74 ± 10.21	-0.84 ± 14.07	0.97
HDL (mmol.l^{-1})	1.93 ± 9.01	1.98 ± 11.36	0.98
LDL (mmol.l^{-1})	1.75 ± 16.64	-1.38 ± 20.13	0.49
Triglycerides (mmol)	-8.84 ± 26.77	1.21 ± 35.82	0.20
Ferritin (μg.l^{-1})	-6.63 ± 43.56	2.48 ± 31.88	0.34
IGF1 (μg.ml^{-1})	6.23 ± 35.68	0.98 ± 19.37	0.47
IGFBP3 (μg.ml^{-1})	7.60 ± 28.94	-3.05 ± 11.74	0.06
IGF1/IGFBP3	-1.49 ± 16.71	4.35 ± 16.40	0.15
Glucose (mmol.l^{-1})	1.06 ± 7.73	1.95 ± 10.05	0.69
Insulin (pmol)	7.19 ± 31.54	2.50 ± 36.15	0.57
QUICKI	-0.38 ± 6.77	0.41 ± 7.08	0.64
HOMA-IR (M.U)	9.02 ± 33.84	5.12 ± 41.91	0.67

Data are presented as: mean ± SD. HIIT = high-intensity interval training; MICT = moderate-intensity continuous training; HDL = high-density lipoprotein; LDL = low-density lipoprotein; IGF-1 = insulin-like growth factor-1; IGFBP-3 = insulin-like growth factor binding protein-3; QUICKI = quantitative insulin-sensitivity check index; HOMA = homeostatic model assessment for insulin resistance; M.U = mass units.

Table S4: Delta changes of skeletal muscle mitochondrial content in obese older adults following 12 weeks of High-Intensity Interval Training (HIIT) and Moderate-Intensity Continuous Training (MICT)

Parameters	HIIT (n=11)	MICT (n=14)	p-value
Skeletal muscle mitochondrial content			
OPA1	38.57 ± 120.49	25.69 ± 70.21	0.75
TFAM	36.25 ± 51.52	57.24 ± 53.88	0.35
VDAC	22.21 ± 101.56	54.53 ± 109.05	0.47
MFN1	39.33 ± 95.08	89.60 ± 210.04	0.47
MFN2	71.57 ± 96.07	104.61 ± 354.75	0.77
DRP1	41.78 ± 167.70	25.92 ± 133.93	0.38
TOM20	54.14 ± 60.23	47.73 ± 111.66	0.87
PARKIN	42.32 ± 63.07	24.92 ± 36.64	0.44
OXPHOS-CI (NDUFB8)	63.08 ± 136.76	76.31 ± 207.62	0.86
OXPHOS-CII (SDHB)	30.74 ± 38.23	74.67 ± 185.59	0.45
OXPHOS-CIII (UQCRC2)	-5.32 ± 37.41	108.34 ± 208.71	0.09
OXPHOS-CIV (MTCO1)	57.38 ± 87.65	166.36 ± 628.06	0.57
OXPHOS-ATPs	-9.57 ± 21.64	119.79 ± 214.30	0.06
OXPHOS-TOT	14.83 ± 39.87	21.59 ± 60.5	0.75

Data are presented as: mean ± SD. HIIT = high-intensity interval training; MICT = moderate-intensity continuous training; A.U = arbitrary unit. OPA1= optic atrophy-1; TFAM = transcription factor A mitochondrial; VDAC = voltage-dependant anion channel; MFN1 = mitofusin-1; MFN2 = mitofusin-2; DRP1 = dynamin-related protein 1; TOM20 = translocase of outer membrane 20; PARKIN = parkin RBR E3 ubiquitin protein ligase; OXPHOS-C = oxidative phosphorylation complex; NDUFB8 = NADH: ubiquinone oxidoreductase subunit B8; SDHB = succinate dehydrogenase complex iron sulfur subunit B; UQCRC2 = ubiquinol-cytochrome C reductase core protein 2; MTCO1 = mitochondrially encoded cytochrome C oxidase I; ATPs = adenosine triphosphate synthase; TOT = Total.
